# Supplementary material for: Barriers and facilitators of fetal heart monitoring with a mobile cardiotocograph (iCTG) device in underserved settings: An exploratory qualitative study from Tanzania
Source: PLoS One. 2024 Dec 5;19(12):e0314812. doi: 10.1371/journal.pone.0314812 (PMC11620659; doi:10.1371/journal.pone.0314812)
Supplement: S1 Table — (DOCX) [file pone.0314812.s001.docx]

**S1 Table Analysis codebook for Barriers and Facilitators of fetal heart monitoring with iCTG**

| **Codes** | **Sub-themes** | **Themes** | **Major groups** |
| --- | --- | --- | --- |
| - Not knowing the iCTG - Seen in a private hospital - No previous experience of using CTG - Never used CTG before - Not knowing how to use - Language barrier | Inadequate knowledge and experience about CTG | Individual related barriers | Barriers |
| - No equipped ambulances - Having non-equipped ambulances - No standby ambulances in health centres - Poor maintenance of ambulances - Lack of fuel for transfer of patients - Ambulance problems - Long distance transfers - Poor roads | Inadequate and poor infrastructures for referrals | Institutional related barriers |  |
| - Paper-based referral letters - Use of personal device for communication - Communication through phone calls - No proper channels for sharing information - No system for sharing information - Internet instability | Unfriendly referral communication platforms |  |  |
| - Shortage of staff - Lack of equipment - No protocols | Inadequacy of human and non-human resources |  |  |
| - Skipping follow up visits - Not seeking medical check up - Less ANC attendance | Illiteracy regarding ANC check-up | Community related barriers |  |
| - Ignorance and fear - Resistant of families to accept a referral decision - Starting ANC late | Myth and misconceptions surrounding childbirth |  |  |
| - Learned about CTG in university - Easy to use - Easy to move - Easy to detect fetal well being - Facilitate quick decisions - Prevent hypoxia related complications - Screening fetal condition - Early identification of fetal hypoxia | Awareness about CTG and its benefits | Motives and desire to use iCTG for FHR monitoring | Facilitators |
| - A wish to receive on-the-job training - Desire to have a reliable FHR monitor - Wishing to have CTG - Wishing to use CTG - Readiness to receive training on CTG use - Desire to continuous FHR monitor | Need and readiness to receive training and use CTG |  |  |
| - Conduct of CEmONC training to HCPs - Provision of mentorship to RCH staff - Intra-facility training | On-the-job training and supportive supervision | Availability of support systems to improve care in primary health facilities |  |
| - CHWs remind mothers to come - CHWs identify pregnant women - Escort women for delivery | Use of community health workers |  |  |
| - Coming for checkup to know fetal condition - Desire to hear the fetal heartbeats sound - Knowledge on birth preparedness | Receiving ANC services from healthcare facilities | Community trust in healthcare system |  |
| - Mentorship - On-the-job training | Capacity building for healthcare providers | Potential solutions to the identified barriers |  |
| - Stationing ambulances in areas with referral challenges | Improving referral system |  |  |
| - Use of volunteers - Employment from the government - Task sharing | Ensure adequate human resource |  |  |
| - Health education - Community outreach - Use of Community Health workers for sensitization | Raise community awareness |  |  |
