# Supplementary material for: Barriers and facilitators of fetal heart monitoring with a mobile cardiotocograph (iCTG) device in underserved settings: An exploratory qualitative study from Tanzania
Source: PLoS One. 2024 Dec 5;19(12):e0314812. doi: 10.1371/journal.pone.0314812 (PMC11620659; doi:10.1371/journal.pone.0314812)
Supplement: S1 Checklist — (DOCX) [file pone.0314812.s002.docx]

**Consolidated criteria for reporting qualitative studies (COREQ): 32-item checklist**

| **No. Item** | **Guide questions/description** | **Section each item has been reported** |
| --- | --- | --- |
| **Domain 1: Research team and reﬂexivity** |  |  |
| *Personal Characteristics* |  |  |
| 1. Inter viewer/facilitator | Which author/s conducted the interview or focus group? | The researchers DLM, MS, YK and RAs |
| 2. Credentials | What were the researcher’s credentials? E.g. PhD, MD | MSc. Midwifery, MSc., PhD |
| 3. Occupation | What was their occupation at the time of the study? | Methods section |
| 4. Gender | Was the researcher male or female? | Female |
| 5. Experience and training | What experience or training did the researcher have? | More than 5 years working experience with a qualitative research experience (methods section) |
| *Relationship with participants* |  | No relationship |
| 6. Relationship established | Was a relationship established prior to study commencement? | Yes |
| 7. Participant knowledge of the interviewer | What did the participants know about the researcher? e.g. personal goals, reasons for doing the research | The study participants were informed about the researcher’s plan, experience, professional background, and reasons for doing the research. (Ethics statement) |
| 8. Interviewer characteristics | What characteristics were reported about the interviewer/facilitator? e.g. Bias, assumptions, reasons and interests in the research topic | Due to the researcher DLM’s profession as a midwife, she worked with other two researchers of different profession and RAs to conduct interviews with healthcare providers. (study strengths and limitations) |

| **Domain 2: study design** |  |  |
| --- | --- | --- |
| *Theoretical framework* |  |  |
| 9. Methodological orientation and Theory | What methodological orientation was stated to underpin the study? e.g. grounded theory, discourse analysis, ethnography, phenomenology, content analysis | Exploratory qualitative study  (Study design) |
| *Participant selection* |  |  |
| 10. Sampling | How were participants selected? e.g. purposive, convenience, consecutive, snowball | Purposive sampling  (study participants and recruitment) |
| 11. Method of approach | How were participants approached? e.g. face-to-face, telephone, mail, email | Face to face |
| 12. Sample size | How many participants were in the study? | Healthcare providers 17, pregnant women 35 and postnatal mothers 25 (Table 1 study participants) |
| 13. Non-participation | How many people refused to participate or dropped out? Reasons? | None |
| *Setting* |  |  |
| 14. Setting of data collection | Where was the data collected? e.g. home, clinic, workplace | Secure and private rooms at the healthcare settings (data collection) |
| 15. Presence of non-participants | Was anyone else present besides the participants and researchers? | No |
| 16. Description of sample | What are the important characteristics of the sample? e.g. demographic data, date | Data were collected between November 2022-June 2023.  The description of a sample is reported in study participants and recruitment and the results (demographic characteristics of study participants) |
| *Data collection* |  |  |
| 17. Interview guide | Were questions, prompts, guides provided by the authors? Was it pilot tested? | Interview guides were developed by the authors. They have been uploaded as supplementary materials. |
| 18. Repeat interviews | Were repeat inter views carried out? If yes, how many? | No |
| 19. Audio/visual recording | Did the research use audio or visual recording to collect the data? | Audio-recording |
| 20. Field notes | Were ﬁeld notes made during and/or after the interview or focus group? | During and after interviews |
| 21. Duration | What was the duration of the interviews or focus group? | Interviews 45-60mins  FGDs 60-110mins |
| 22. Data saturation | Was data saturation discussed? | Yes, we used information power.  Page 9 (data collection) |
| 23. Transcripts returned | Were transcripts returned to participants for comment and/or correction? | No |
| **Domain 3: analysis and ﬁndings** |  |  |
| *Data analysis* |  |  |
| 24. Number of data coders | How many data coders coded the data? | Four  Page 10 (data analysis) |
| 25. Description of the coding tree | Did authors provide a description of the coding tree? | Yes (described as codebook in this paper)  Page 10 |
| 26. Derivation of themes | Were themes identiﬁed in advance or derived from the data? | Derived from data.  Page 10 |
| 27. Software | What software, if applicable, was used to manage the data? | Dedoose  Page 10 |
| 28. Participant checking | Did participants provide feedback on the ﬁndings? | Yes  Page 11 |
| *Reporting* |  |  |
| 29. Quotations presented | Were participant quotations presented to illustrate the themes/ﬁndings? Was each quotation identiﬁed? e.g. participant number | Yes (results section) |
| 30. Data and ﬁndings consistent | Was there consistency between the data presented and the ﬁndings? | Yes |
| 31. Clarity of major themes | Were major themes clearly presented in the ﬁndings? | Yes  Page 13-18 |
| 32. Clarity of minor themes | Is there a description of diverse cases or discussion of minor themes? | We discussed the sub-themes in each major theme |
