# Supplementary material for: Barriers and facilitators of fetal heart monitoring with a mobile cardiotocograph (iCTG) device in underserved settings: An exploratory qualitative study from Tanzania
Source: PLoS One. 2024 Dec 5;19(12):e0314812. doi: 10.1371/journal.pone.0314812 (PMC11620659; doi:10.1371/journal.pone.0314812)
Supplement: S1 File — (DOCX) [file pone.0314812.s003.docx]

1. **Interview guide for District Medical Officer and District Reproductive and Child Health Coordinator**
2. **Demographic Characteristics**

| Participant Code: | District: |
| --- | --- |
| Position: | Age: |
| Cadre: | Sex: |
| Professional education level: | Marital status: |
| Working experience (years): | Years in a leadership position: |
| Name of Interviewer: | Date of interview: |

1. **Interview questions and probes**
2. First of all, thank you very much for your time. We would like to start by getting your experience with the antenatal and childbirth care services provided to pregnant women at health facilities in this district.

Probes: antenatal care services coverage; ways to examine fetal condition during antenatal check-up and labour; challenges for current fetal monitoring; adequacy of healthcare providers.

1. Thank you for your explanations. If we were to implement the technologies for examining fetal heartbeats using iCTG in primary health care facilities, what do you consider facilitators for successfully implementing this system?

Probes: training, infrastructural support, friendliness, adequacy of staff, availability of supportive supervision, users and community acceptance, competence of HCPs, willingness among HCPs

1. What could be the main barriers to implementing this technology?

Probes: complexity, sufficient knowledge and skills among staff, shortage of staff, user’s adherence, limited infrastructure, acceptability by the HCPs, users, and community acceptance, etc.

1. As we finalize the interview, is there anything you would like to say that is related to this topic we have not discussed?
2. **Interview guide for healthcare providers**
3. **Demographic Characteristics**

| Participant Code: | Health Facility: |
| --- | --- |
| Cadre: | Age: |
| Professional education level: | Marital status: |
| Working experience (years): | Sex: |
| Name of Interviewer: | Date of interview: |

1. **Interview questions and probes**
2. First of all, thank you very much for your time. We would like to start by getting your experience with the provision of antenatal care and childbirth services to pregnant women in this health facility.

Probes: weeks of pregnancy at first antenatal booking for most women, what motivates them to come for ANC, reasons for those who do not come for ANC, how much time does it take for women to wait for services, how much does it cost, how many deliveries per month, referrals, main reasons for referrals to secondary health facilities, etc.

1. How do you check or examine the fetal condition? Probes: equipment for prenatal check-up, devices used to listen to fetal heartbeats, ways to detect fetal heartbeat abnormalities, awareness or knowledge about CTG, etc.
2. How often do you conduct health education sessions for pregnant women?

Probes: number of sessions per week, ways used to deliver health education, content of sessions, use of guidelines, knowledge about the use of mobile health.

1. In this facility, if we were to implement digital technology for examining fetal heartbeats using iCTG what would be the main facilitators and barriers to successful implementation? Probes: training, infrastructural support, friendliness, adequacy of staff, availability of supportive supervision, users and community acceptance, competence of HCPs, willingness among HCPs
2. Is there anything you would like to say related to this topic that we have not discussed?
3. **A focus group discussion guide for pregnant women and postnatal mothers**

**A. Demographic information of participants**

| **S/N** | **Age (years)** | **Education level** | **Occupation** | **Number of pregnancies** | **Parity** | **Number of ANC visits** |
| --- | --- | --- | --- | --- | --- | --- |
| 1 |  |  |  |  |  |  |
| 2 |  |  |  |  |  |  |
| 3 |  |  |  |  |  |  |
| 4 |  |  |  |  |  |  |
| 5 |  |  |  |  |  |  |

**B. Focus group discussion questions**

1. First of all, I would like us to start our discussion by hearing your experience with how you realized that you were pregnant. Please tell us how you found out that you are pregnant. Probes: average number of weeks, reasons for late detection, actions taken after realizing a pregnancy status, how long did you wait to start an antenatal check-up, what made you decide to go for your first contact, awareness of danger signs, and the main challenges pregnant women face during pregnancy.
2. How do you choose where to go for antenatal checkups and childbirth services? Probes: availability of services (ultrasound, non-stress test, intensive fetal heart rate monitoring, adequate number of providers, provider’s attitudes); how do you go to the health facility; how long does it take to go to the hospital; who accompanies you.
3. What are the challenges for those who do not go for antenatal care checkups or childbirth services in health facilities? Probes: lacking preferred services, distance, costs, health facility-related factors.
4. If we introduce a digital device for monitoring fetal heart rate and detecting fetal heartbeat abnormalities, it will aid healthcare providers in prompt intervention. How would you like this service to be provided? Probes: What are the barriers to the uptake of the service? What can be the facilitators for women to utilize the service?
5. As we finalize our discussion, I would like us to discuss the strategies that can be used to ensure that pregnant women utilize childbirth services in healthcare facilities.
6. Is there anything important related to today’s topic that we have not discussed?
