# Supplementary material for: Barriers and facilitators of fetal heart monitoring with a mobile cardiotocograph (iCTG) device in underserved settings: An exploratory qualitative study from Tanzania
Source: PLoS One. 2024 Dec 5;19(12):e0314812. doi: 10.1371/journal.pone.0314812 (PMC11620659; doi:10.1371/journal.pone.0314812)
Supplement: Attachment — Submitted filename: review for article.docx [file pone.0314812.s005.docx]

Drear chef editor

Thank you for the opportunity to review manuscript entitled” Barriers and facilitators for implementing fetal heart rate monitoring with a mobile cardiotocogram device (iCTG) in underserved settings: An exploratory qualitative study from Tanzania “

Below are my comments for this article:

1- Lack of Novelty.
2- It seems that the researchers could not justify why a qualitative study is needed.
3- One of the strengths of this study could be providing a solution, which is not present in the results.
4- In the results section, it is not clear how many codes, initial categories and sub categories have been obtained.
5- It seems that the results do not add anything new to science. We are faced with the realities of using this device. Health service providers seem to know these facts.

Kind regards

Dr ghaffari
