# Supplementary material for: Barriers and facilitators of fetal heart monitoring with a mobile cardiotocograph (iCTG) device in underserved settings: An exploratory qualitative study from Tanzania
Source: PLoS One. 2024 Dec 5;19(12):e0314812. doi: 10.1371/journal.pone.0314812 (PMC11620659; doi:10.1371/journal.pone.0314812)
Supplement: Attachment — Submitted filename: Response to Reviewers revised.doc [file pone.0314812.s006.doc]

**Point by Point Responses to the Reviewer’s Comments**

| **S/N** | **Comment** | **Response** | **Page and lines changes inserted** |
| --- | --- | --- | --- |
| **Reviewer 1** | | | |
| 1 | Lack of Novelty. | Thank you for this comment.  We might not be very clear about the novelty of this study.  To make it clearer, we improved the writing to highlight the literature gap and elaborated the novelty of this study by indicating the need to understand the barriers and facilitators to implement the FHR monitoring innovations in the undeserved countries such as Tanzania in the introduction section.  In addition, we show that this is among the few qualitative studies that explored the barriers and facilitators of implementing a mobile cardiotocograph device in primary care settings in Tanzania in the discussion section. | Page 4-5, Lines 96-111  Page 18, Lines 428-430 |
| 2 | It seems that the researchers could not justify why a qualitative study is needed. | Thank you for raising this concern.  Qualitative study plays a crucial role in decision-making before implementing a strategy. They provide in-depth insights into different perspectives of stakeholders, which quantitative data alone may not capture. It helps to identify potential barriers, facilitators, understand contextual factors, and uncover hidden needs, ultimately leading to more informed and effective implementation strategies.  We have added the justification of why exploratory qualitative study was needed before we introduce the use of iCTG in health facilities. | Page 5, Lines 112-126 |
| 3 | One of the strengths of this study could be providing a solution, which is not present in the results. | Thank you for this comment.  The study provides insights on the things to be considered before implementation of the strategy in this context leveraging to the identified facilitators. The need and readiness to receive training and use iCTG and the available system to provide on-the job training could be used as possible solutions to fill the knowledge and skills gap. Furthermore, the availability of community health workers and the community trust to receiving ANC services are possible solutions to raising awareness and correcting myths through delivery of health education.  To be clearer to the readers, we added this information in the discussion of the specified results mentioned above and in the study strengths section. | Page 19, Lines 448-450  Page 20, Lines 484-487  Page 21, Lines 492-496 |
| 4 | In the results section, it is not clear how many codes, initial categories and subcategories have been obtained. | Thank you for this observation.  In this study, we used thematic analysis. Braun and Clarke (2006) thematic approach analyse the underlying patterns (themes and meanings) from the shared experiences, offering a richer and more nuanced understanding of the context and the participants’ perspectives.  Therefore, we presented the codes, sub-themes and themes generated during analysis. We have indicated the number of codes, subthemes and themes in the paragraph describing the themes emerged during analysis. | Page 9-10, Lines 215-235  Page 12, Lines 268-270 |
| 5 | It seems that the results do not add anything new to science. We are faced with the realities of using this device. Health service providers seem to know these facts. | Thank you for raising this concern.  This study aimed to analyse the barriers and facilitators on the implementation of iCTG specifically on primary healthcare facilities to inform the development of intervention and training for healthcare providers.  The results presented in this paper add merit to the science in the fact that, we describe why we needed evidence for implementation barriers and facilitators to determine strategies for optimal use of iCTG in limited resource settings. In addition, by reading the findings of this paper,  future implementors would benefit by identifying the available context specific opportunities/strategies to support the implementation of evidence-based interventions in the healthcare system despite the faced challenges/realities. | Page 12-18, Lines 267-423 |
| **Reviewer 2** | | | |
| 1 | Page 5 paragraph 2 live 8 should read thus "Despite both district hospital..................... , the 2 hospitals functions as referral | Thank you for the suggestion.  We have revised the sentence. Now reads as *“Despite both the district hospital and the health center falling within the primary level of the pyramidal Tanzanian health service delivery system, the district hospital functions as a referral hospital for their immediate health facilities due to their greater capacity to provide comprehensive emergency obstetrics and newborn care.”* | Page 6, Lines 134-137 |
| 2 | How many days did the collection of information take. The recording for the health workers lasted 45 to 60 mins each | Thank you for this comment.  We used a total of 25 days for data collection; however, we spent more days in the file for other procedures including contacting hospital administrators, recruitment and setting up appointment for interviews and FGDs.  We revised the data collection section to include the information on the number of days spent for data collection and edited the last sentence about the time used to conduct interviews. Now reads as “The interviews lasted between 45 and 60 minutes”. | Page 8, Lines 189-192  Page 9, Lines 202-203 |
| 3 | Was the questionnaire for the health workers administered by a single researcher or more than one. | Thank you for raising this concern.  The data collection team was composed of five people (3 researchers and 2 research assistants). Each session of interview or FGD was conducted by a pair of two people. One asking questions and another taking notes and recording the session. At this moment, other people waited outside the room and joined the interviewing team during the debriefing to hear what was transpired during the interview/FGD. Then, the following interview was done by a different team.  We included this information in the methods section. | Page 8, Lines 184-194 |
| 4 | The recording of the interview is a positive development and making of transcript by more than one person gives credence to information. | Thank you for this information which complement to what we observed.  We audio-recorded all interview and discussions to ensure that all participant’s accounts are captured. The transcripts produced were made from more than one person. The maximum use of participants’ accounts and the use of more than one data collector ensured the credibility of the data.  We included this information in the methods section. | Page 11, Lines 244-247 |
| **Reviewer 3** | | | |
| 1 | What is the cost of the iCTG device and how do you think this could be sustainable in Tanzania. | Thank you for raising this concern.  There are various types of iCTG based on their functions. For example, the common type of iCTG has three primary functions: (1) monitoring FHR; (2) monitoring contractions; and (3) sharing data via the internet. So, if the context-specific requirement does not require the function of sharing data due to any reason or due to internet challenges, then iCTG can be customized with only two major functions: monitoring fetal heart rate and contractions. And this makes the purchase price lower compared to the original price. The one discussed in this paper has all three functions and costs about 4.5 million Tanzanian shillings. However, since the company is currently working on reducing the price, we did not include the information in the manuscript.  If our study shows positive health impacts in Tanzania, the results will provide strong evidence and valuable lessons learned for sustainability of this innovative healthcare technologies to save lives, impact the quality of care, and enable women experience a positive childbirth experience. | N/A |
| 2 | How could it be useful for women considering the limiting factors you found "individual related that include inadequate knowledge and skills to use iCTG, institutional related barriers attributed to limited referral infrastructures and shortage of staff, and community related barriers such as myths and misconceptions that limited the number of antenatal care checkups" | Thank you for asking.  The identified barriers and facilitators will help the researchers to design the intervention and prepare for implementation.  This included: the provision of training to the healthcare providers to fill out the gap of knowledge and skills to use the iCTG. To raise awareness among consumers, whereby community health workers will be used to sensitize women to attend antenatal care. But also, we developed a smartphone app with educative contents for midwives to use during the provision of health education to pregnant women as well as informing them about the benefits of having regular antenatal checkups. Therefore, women will eventually enjoy the benefits of good perinatal healthcare from the health facilities utilizing iCTG.  We included this point in the discussion. | Page 21-22, Lines 488-514 |
| 3 | How could it be a primary choice to invest in Tanzania, rather than increase facilities and remove barriers to access primary care rooms. | Thank you for your concern.  We acknowledge that the use of iCTG may be the primary choice to invest at least at the moment in Tanzania. This is because, improving the situation is not limited to increasing facilities and removing barriers to access primary care. Tanzania is facing a critical shortage of human resource for health; this necessitates the implementation of suitable technologies/innovations for healthcare delivery as one of the solutions to this problem as well to improving the health outcomes.  Reducing maternal and neonatal mortality is still a priority agenda in Tanzania. Currently, the neonatal mortality is at 24 deaths per 1,000 live births, which is much higher than the one indicated in SDG 3. Intensive fetal monitoring during late pregnancy and in labour plays an important role in detecting perinatal hypoxia early and saving lives. The iCTG portable device has been recommended as a suitable strategy to ensure perinatal safety in settings with medical resource challenges including shortage of staff.  We included this information in the introduction. | Page 3, Lines 65-70  Page 4, Lines 84-92 |
