# Supplementary material for: Barriers and facilitators of fetal heart monitoring with a mobile cardiotocograph (iCTG) device in underserved settings: An exploratory qualitative study from Tanzania
Source: PLoS One. 2024 Dec 5;19(12):e0314812. doi: 10.1371/journal.pone.0314812 (PMC11620659; doi:10.1371/journal.pone.0314812)
Supplement: Attachment — Submitted filename: I Ctg review.docx [file pone.0314812.s007.docx]

Thank you for letting me review your paper. The research question is interesting to the Journal’s audiences.

There are multiple typos, spelling errors, grammatical errors, poor word usage, punctuation, and formatting problems. These must be addressed to fulfill the standards of the journal. The language should undergo proofreading by a native or get professional English editing services.

To mention a few … lines 85, 86, 89, 90, 96,103, 144, 147, 233 typo errors, punctuation….

The AI score of the study is 5%, which is commendable

The plagiarism score is 13%, which indicates further rephrasing is required to keep it below 10%

CTG stands for cardiotocography, which involves monitoring the heart rate (cardio) and uterine contractions (toco). However, the manuscript seems to focus only on heart rate, with no mention of uterine contractions throughout the paper (It was mentioned only once, line 88). It is unclear to refer to it as CTG when it is merely Doppler due to this error. It is imperative that this limitation be addressed in the section on limitations of the study.

What was the professional level of the individual who conducted IDI? How was bias mitigated in the selection, of the women, as you mentioned that the on-call midwife was in charge of selecting and organizing the pregnant mothers? Could there be a bias to choose inherently pregnant mothers who are satisfied with the overall care including the CTG? Who selected the interviewees, if the midwife was only, limited to selecting a private place only?

The FGDs were conducted by the investigators, DLM, and co. What measures were taken to decrease bias? Was there any bracketing? it needs to be mentioned in the methods section. Other than your statement ‘’generated inductively using thematic analysis and presented with the support of subthemes and succinct quotes’’

Institutional Barriers

I am having trouble relating these findings, especially about the study's objective. Are you trying to say that health professionals do not use CTG because anomalies in the referral system prevent them from sending patients when they detect fetal heart abnormalities?

Community-related barriers,

The FGD should have focused on i CTG instead of exploring the whole health system. Is the long queue due to the usage of i CTG? You need to clearly address it in the discussion section.
