# Supplementary material for: Barriers and facilitators of fetal heart monitoring with a mobile cardiotocograph (iCTG) device in underserved settings: An exploratory qualitative study from Tanzania
Source: PLoS One. 2024 Dec 5;19(12):e0314812. doi: 10.1371/journal.pone.0314812 (PMC11620659; doi:10.1371/journal.pone.0314812)
Supplement: Attachment — Submitted filename: Response to Reviewers.doc [file pone.0314812.s008.doc]

**Responses to Reviewers**

| **S/N** | **Comment** | **Response** | **Page and lines changes inserted** |
| --- | --- | --- | --- |
| **Academic editor** | | | |
| 1 | The conclusions must be drawn appropriately based on the data presented. | Thank you for the comment.  We revised the conclusion for both the abstract and the main paper. Now, it corresponds with what is presented in the data/results. | Page 2-3, Lines 51-58 |
| 2 | The language edition of the manuscript should be made by native English speaker. | This is well noted.  The language editing has been done to correct grammar errors by a native English speaker. | Throughout |
| 3 | In your manuscript you give main focus only on heart rate, with no mention of uterine contractions throughout the paper seems a limitation, please check if you can incorporate it briefly on the limitation section | Thank you for raising this concern.  In this phase, we mainly focused on the use iCTG to monitor fetal heart rate during late pregnancy to improve pregnancy and childbirth outcomes. The later phase will assess the use of iCTG in the labour room and incorporate the component of contractions. We have provided a brief explanation in the limitation section. | Page 23, Lines 523-526 |
| **Reviewer 4** | | | |
| 1 | The whole paper needs a grammar edition. | Thank you for your concern.  We have gone through the manuscript and corrected grammatical errors accordingly. | Throughout |
| 2 | This is a good entry point for identifying the obstacles before any implementation research is done so that some problems may be fixed beforehand and correctly. But there are a lot of barriers identified and it is worth mentioning the solutions in summary or table or figure which are listed by the participants and may help in addressing the root cause during the implementation. | Thank you for raising this concern.  We have provided a table (Table 2) in the results section that shows the potential solutions reported by the participants. | Page 18-19, Lines 426-427 |
| 3 | Page 3, Line 70-72, I think this is a vague meaning and no clear meaning of mentioning this in this paper's context or must be with a reason why not effective if there is access to them? What makes the difference in safety, appropriateness, WHY? | Thank you for this observation.  We have revised the sentence. Now reads as ‘*Many medical devices designed for use in high-income countries have several limitations when reciprocated in low-resource settings. [8, 9]. Currently, technological innovations are being leveraged to ensure the availability of suitable and appropriate devices for monitoring fetal surveillance in LMICs [8]’.* | Page 3, Lines 70-73 |
| 5 | Issue of feasibility and sustainability iCTG use in Tanzania? | Thank you for raising this concern.  Our feasibility check indicated that the use of iCTG in Tanzania was feasible, needed and acceptable. The sustainability will depend on our results after the implementation. If our study shows positive health impacts in Tanzania, the results will provide strong evidence and valuable lessons learned for sustainability of this innovative healthcare technologies to save lives, impact the quality of care, and enable women experience a positive childbirth experience. | N/A |
| 6 | Shortage of staff is one of your barriers and you mentioned it as a solution for the use of iCTG. Does it contradict? Especially for those who receive the record and interpret and decide and intervene (GYN/OBS specialists) | Thank you for raising this concern.  One innovative part of iCTG is that it doesn’t require continuous provider attendance during measurement compared to an intermittent fetal monitor which requires constant provider attendance to a patient. Thus, it is considered suitable in settings with resource challenges, including a shortage of staff.  We acknowledge that our findings contradict what we report as a solution. However, participants (providers) perceived the staff shortage as a barrier to using iCTG because they considered a 20-minute measurement amidst a long queue of pregnant women waiting to receive service from a single provider a problem. Moreover, there was a limited number of OBGY specialists to receive the recordings, interpret them, and make decisions. | N/A |
| 7 | Issue of Referral is a big barrier vs the use of iCTG? How are you going to negotiate this? | Thank you very much for this concern.  Ambulances were stationed in zones/areas with referral issues, which helped to overcome the referral barrier. The authors have included a table of the proposed solutions to the identified barriers in the results section. | Page 18-19, Lines 426-427 |
| **Reviewer 5** | | | |
| 1 | There are multiple typos, spelling errors, grammatical errors, poor word usage, punctuation, and formatting problems. These must be addressed to fulfill the standards of the journal. The language should undergo proofreading by a native or get professional English editing services. To mention a few … lines 85, 86, 89, 90, 96,103, 144, 147, 233 typo errors, punctuation…. | Thank you for this valuable comment.  The language editing has been done to correct grammar errors by a native English speaker. | Throughout |
| 2 | The AI score of the study is 5%, which is commendable The plagiarism score is 13%, which indicates further rephrasing is required to keep it below 10% | Thank you for this valuable comment.  We have done further rephrasing which might reduce the percentage of plagiarism. | Throughout |
| 3 | CTG stands for cardiotocography, which involves monitoring the heart rate (cardio) and uterine contractions (toco). However, the manuscript seems to focus only on heart rate, with no mention of uterine contractions throughout the paper (It was mentioned only once, line 88). It is unclear to refer to it as CTG when it is merely Doppler due to this error. It is imperative that this limitation be addressed in the section on limitations of the study. | Thank you for raising this concern.  The iCTG device can monitor both fetal heart rate and contractions, however, this study only focused on implementing iCTG during late pregnancy to improve fetal outcomes. Nevertheless, the later phase of the study focused on the use of iCTG during labour and incorporated the components of monitoring fetal heart rate and contractions.  We have addressed this concern in the limitation section. | Page 23, Lines 523-526 |
| 4 | What was the professional level of the individual who conducted IDI? How was bias mitigated in the selection, of the women, as you mentioned that the on-call midwife was in charge of selecting and organizing the pregnant mothers? Could there be a bias to choose inherently pregnant mothers who are satisfied with the overall care including the CTG? Who selected the interviewees, if the midwife was only, limited to selecting a private place only? | Thank you very much for asking.  Regarding the individuals who conducted IDI; one had a bachelor’s degree, and one had a master’s degree.  The midwife was not involved in the selection of study participants. Rather, she introduced the researcher to women who were available at the clinic. The selection of the eligible participants was solely conducted by the data collection team. | Page 8, Lines 186-188  Page 7, Lines 154-158 |
| 5 | The FGDs were conducted by the investigators, DLM, and co. What measures were taken to decrease bias? Was there any bracketing? it needs to be mentioned in the methods section. Other than your statement ‘’generated inductively using thematic analysis and presented with the support of subthemes and succinct quotes’’ | Thank you for asking.  We relied on the following methods to reduce bias:   1. Triangulation of researchers. We had more than one interviewers. 2. Triangulation of study participants. We collected data from both supply and demand side. And we ensured maximum variation among study participants. 3. Triangulation of data coders. We employed a team-based approach for coding. 4. Checking for coding reliability. At first, we both coded the same transcript, and then we convened to resolve the discrepancies and disagreements that emerged after coding same transcript before we continued with other transcripts. 5. Lastly, researchers not being seniors or early career made participants to be more comfortable. | Page 11, Lines 245-256 |
| 6 | Institutional Barriers I am having trouble relating these findings, especially about the study's objective. Are you trying to say that health professionals do not use CTG because anomalies in the referral system prevent them from sending patients when they detect fetal heart abnormalities? | Thank you for raising this concern.  This qualitative study was done to identify potential barriers, facilitators, understand contextual factors, and uncover hidden needs, that could lead to more informed and effective implementation strategies. Identifying the obstacles helped in designing intervention and fixing some problems beforehand.  Our findings indicated that, the referral system was a barrier in case there was a need for referral after the detection of abnormal FHR with iCTG. Nevertheless, this was not a barrier for healthcare providers not to use the iCTG rather, it was foreseen as a barrier only in the need of a referral following the results of iCTG recording.  Therefore, the potential solution to this obstacle was to station the ambulances in the zones/areas which had referral system challenges. | Page 5, Lines 114-125  Page 18-19, Lines 426-427 |
| 7 | Community-related barriers, The FGD should have focused on iCTG instead of exploring the whole health system. Is the long queue due to the usage of iCTG? You need to clearly address it in the discussion section. | Thank you for this comment.  Before the implementation of iCTG we wanted to explore the need from the demand side on readiness towards utilization of the service. Thus, we went further to interviewing pregnant women. From the interviews, it came out that illiteracy regarding ANC checkup might affect the utilization of the service. Some women reported defaulting to follow-up checkups as a result of the long queue for antenatal care services in healthcare facilities. | Page 21, Lines 478-490 |
